# Certain Diseased Conditions of the Ovum and Its Envelopes, Induced by Constitutional Vice in the Mother, as a Cause of Abortion

**Published:** 1861-01

**Authors:** C. A. Logan

**Affiliations:** Leavenworth City, Kansas


### *Certain Diseased Conditions of the Ovum and its Envelopes, induced by Constitutional Vice in the Mother, as a cause of Abortion.* By C. A. Logan, M.D., Leavenworth City, Kansas.*

The deep obscurity in which the mysterious process of generetion still lies hidden, renders it impossible for us to take cognizance of the conditions under which the germ may expand into a healthy, living being, or degenerate into a blighted mass. The light, however, which has been thrown upon it in recent years by the labors of such men as Barry, Von Baer, Kolliker, Weber, Sharpey, Coste, Lee, J. Reid, and others, has enabled the observing physician to draw many practical deductions, and apply them with advantage to the furtherance of successful gestation. Reliable statistics show, however, that abortion is an accident of very frequent occurrence, and the difficulty of preventing its repitition in many cases, prove conclusively the imperfection of our knowledge.

Mr. Whitehead, in his work on Abortion and Sterility, has given some statistics, which, startling as they appear, are even exceeded, I believe, in the American females. Mr. Whitehead says: "Two thousand married women, in the state of pregnancy, admitted for treatment at the Lying-In Hospital, were interrogated in rotation respecting their existing condition and previous history. Their average age at the time of inquiry was a small fraction below 30 years. The sum of their pregnancies already terminated, was 8681, or 4.38 for each; of which rather less than one in seven had terminated aboitively. But as abortion occurs somewhat more frequently during the latter than in the first half of the

*An Essay read before the Leavenworth Medical and Surgical Association.

child-bearing period, the real average, consequently, will be rather more than one in seven." Again: "Of 747, all had aborted once at least, some oftener. Their average age was 32.08 years. The sum of their pregnancies was 4775, or 6.37; that of their abortions 1222, 1.63 for each person." From this exhibit we discover that more than one woman out of every three aborts before she attains the age of 30 years. These statistics are gathered from a class of women who, experience teaches us, are in one of those extremes of society in which abortions are most apt to occur. Could the same data be obtained among the class of females in this country whose social status corresponds to the above, I am of the opinion that the number of aborting women would be increased.

The causes of abortion are divided by most writers into maternal and ovuline, the latter embracing indefinitely all causes which compromise the life of the child. It is in this division that I believe much harm is done. While the general fact is stated, that the ovule may be diseased within itself, and thereby compromise its own existence, yet it is not taught us that the causes of that condition by which the vitality of the germ is vitiated, must reside in the parents whose product it is. We are thus restrained from looking into the ultimate causation of these difficulties, and content ourselves with knowing that we have done all the books prescribe. In a case of oft-repeated abortion we may have satisfied ourselves that there exists no ulceration or other disease of the cervix—no organic or functional derangement of any of the viscera; we may have carefully fortified our patient against the possibility of nervous shock or excitement, undue effort, of accidents of all kinds; and when holding in our hand the little product of the blighted conception, we discover that by some mysterious disease of the involcra or other fœtal constituents its nutrition has been intercepted, we contentedly solace ourselves and patient with the assurrance that the cause was *ovuline*, and could not be avoided. In all such cases would it not be well for us to go behind the ovule, and inquire why it is that the mother can not eliminate a healthy germ, which shall, in due season, be developed into the equally healthy child? Can there be any doubt that syphilitic or scrofulous depravation of the maternal blood is capable of vitiating the vitality, and setting up various lesions in any and all of the ovular structures? I think not, and am of the opinion that if in many of those obstinate cases, where "the habit of aborting" is acquired, we were to inquire into the constitutional tendencies of the mother, we should find one of these causes in operation, and that the difficulty would give way under a proper constitutional treatment.

That various affections of the fœtal membranes, by which their absorbing and secreting functions are impaired or destroyed, are a prolific source of early abortions, I have repeatedly satisfied myself, by minute examination of the altered consistence and texture of the membranes, not explainable by post-mortem changes. The fact that the fœtal envelopes are subject to disease, is presented to us daily in those cases that, though having gone to the full term, present various abnormal conditions which may seriously impede delivery. Of these may be mentioned preternatural toughness, or friabillity of the membranes; and a largely increased or diminished quantity of fluid; in the former case, perhaps creating uterine intertia—in the latter subjecting the os to the slow dilatation of the presenting part. Nor is the amnion alone liable to these varying conditions of secretion, as the following cases will illustrate:

CASE 1ST.—Aug. 27, 1858., Mrs. L——, taken in labor with the second child. A vaginal examination revealed a head presentation, with a fully dilated os. The stage of labor having been completed, I ruptured the membranes, which were rather tough, and considerable quantity of fluid was discharged. A calm ensued in the pains, and upon their being resumed the head was driven rapidly into the inferior strait, but with the bag of waters still preceding it. I carefully examined the protruding membrane, and satisfied myself that this one had not been ruptured, and pressing my finger against it, a second quantity of water was discharged, when the scalp was felt, and before a great while the child was born.

Case 2nd.—June 29, 1860, Mrs. B.——, brought to bed with her first child. The os underwent a rapid dilatation, and in the absence of pain it was easy to determine that the feet presented. The feature which I wish particularly to notice, however, was the bag of waters as it presented. The finger, when introduced during a pain, first impinged on a membranous bag, rather loose and flabby, but upon being pressed further in it came in contact through the parieties of the first with the hard, unyielding collection in the second bag. I do not know that I can better describe it, than by likening it to a small bladder filled to its utmost capacity with water, put into a large bladder but partially filled. If then the finger should be made to feel the small bladder through the fluid in the larger one, the idea would be pretty accurately conveyed. Recognizing thus two distinct collections, I determined, if possible, to procure the fluid of the first for the purpose of analysis. Obtaining the barrel of a small gutta-percha syringe, and stopping up the lesser aperture, I introduced it along with the index finger of the left hand, while a quill-pen was introduced with the same finger of the right hand. The sac was punctured in such places as to collect the fluid in the syringe, which was then withdrawn, with the finger over it to prevent the escape of the liquid, and set aside for future examination; but, much to my chagrin, a bungling nurse, in her clumsy haste, knocked it over and spilled the contents. A small quantity of liquid escaped from the rupture of the first sac, and a large quantity from the rupture of the true bag. I have frequently read narrations of this double collection of fluid, in the various medical journals, accompanied with the wonderment of the writers as to its production.

Some of our standard authors, as Dewees, Ramsbotham and Chailly barely mention it under the name of the "false waters," without attempting any inquiry into its cause. I am of the opinion that the collection may either exist between the decidua reflexa and the chorion, or between the chorion amnion, in consequence of diseased action in either, whereby a hyper-secretion takes place. That these membranes are subject to inflammation, thickening, opacity, and the effusion of fluid, has been satisfactorily established by M. Mercier. That a syphilitic, scrofulous or tubercular taint may establish such diseases from the first formation of the fœtal envelopes to such an extent as to interfere with the healthy nutrition of the embryo, as to cause its death in the weeks; or, acting less intensely, to simply give rise to a functional lesion, evidenced by the cases narrated, but not sufficient to destroy life, I think is equally plain.

As further evidence of the derangements to which the embryotic membrances are subject, the following case may be related:

CASE 3D.—September 14, 1859, Mrs. J.—— was taken in labor with her first child. When I arrived I found the os almost fully dilated, but there was nothing to be felt, save the hard fœtal head, and I naturally enough supposed the membranes had ruptured the labor progressed, and in a short time the child was born. Upon an examination, however, I found the infant *enveloped in a strong membranous bag, fitting as closely to the body as a glove to the hand, with no drop of intervening fluid.* It was eneire, except where it had been torn from its attachment to the placenta, and so tough that it required considerable effort to tear it open; upon doing which, however, the child cried lustily. The infant was a female, and those of the Irish matrons who stood near and saw the peculiarity, declared that it was born with a *caul* over its face, and was destined to astonish the world by its power of soeing into futurity, when it should reach the proper age. Had it been able to explain to me the mystery of its present situation, I should have been better satisfied.

Here, then, is a case directly opposite of those before related; and what may serve to throw some light upon it and fix it as a vice originating in the maternal economy, is the fact that although the infant was kept for some two or three weeks at intervals pulling at the breast *yet no drop of milk was ever secreted by the mother*, and the child was fed by a humane cow friendly to the cause of distressed infancy. To all appearances the mother would be judged a healthy woman; she had light hair, blue eyes and fair skin, but in childhood had suffered with a unhealthy purulent discharge from the ear, together with some other local manifestations of a scrofulous diathesis. It appears evident to me, that although these cases only establish the fact that the fœtal membranes may be diseased in such a way as to derange their secreting properties yet we are justified in assuming that an intenser form of morbid action, so to speak, may so destroy the integrity of their structure as to incapacitate them from performing their peculiar function, and the embryo perish for want of nourishment.

Some of the authorities mention the fact that constitutional vices in the mother may act as predisposing causes of abortion, but do not lay the stress upon them which I think their importance demands. If a female be affeted with a scrofulous diathesis, a condition which has its scource in the great river of life itself, can the little cell, whose pabulum it is, so elaborate the vital fluid as to cast out its morbific properties, and appropriate only that which is competent to develop it into the strong and vigorous child? Or would we rather except to see it impregnated with the weakness of the element from which it springs, and find the tendency to local inflammation and deranged nutrition generally setting up disease in its vital organs and cutting short its existence?

I believe the fœtal chorion to be an organ of nutrition, performing the same office for the fœtus in the early weeks of pregnancy that the lacteals do at a later time for the independent being. If then the nutritive function of the mother is diseased, as manifesting itself in scrofulosis, the inevitable tendency is to a lesion of the fœtal organs of nutrition, in which the chorion may participate, as well as to a perverted condition of the economy generally. That there does exist this influence by which a scrofulous or other cachectic state of the maternal system strongly induces to disease and death in the ovum, is confimed, in my mind, by a state of things I have noticed since my residence in Kansas. It is well known that a large proportion of our inhabitants have emigrated from the Eastern States, and many of them for the sole purpose of breaking up a disposition to, or of curing a confirmed tuberculosis—a disease which we all know is the scurge of that portion of our country. Whether experience shall hereafter demonstate that this climate is peculiarly adapted to diseases of a tuberculous character and its minor manifestation, scrofula, I can not now speak positively, but I have certainly witnessed a remarkable amendment in many of the above cases that, "having thrown physic to the dogs," could be attributed to nothing save atmospherical influence and increased physical power, induced by more favorable habits of life. Some of these, to come back to the subject, have been females whose early history was linked largely with frequent abortions, but who improved in general health to a remarkable extent, and soon conceiving, have gone to term, in some cases, would almost certainly have induced miscarriage. So frequent is this that the prolific nature of the Kansas women is a frequent laughing topic among themselves. The following case may be given in illustration:

CASE 4TH.—Mrs. K—— removed to Kansas in 1858, from Boston, having been advised, according to her statement, by the celebrated Dr. Bowditch to do so as a means of improving her health, which for ten years previously and particularly the latter eight, being her married period, had been very critical. She had suffed much mennorrhagia; in eight years was pregnant seven times, and had aborted as often; was anemic, debilitated, and supposed to be on the threshold of "a galloping consumption." When I first saw her she was much emaciated; the lips and conjunctiva were bloodless; appetite perverted; bowels excessively constipated, not being moved for a period of two weeks at times; an exacerbation of fever every night. There were several glandular enlargements under the jaw upon either side, and there existed that peculiar brightness of the eye which so characterizes the scrofulous subject. There was a slight cough, with the occasional expectoration of a thin, whitish fluid, but the physical sign did not reveal any of the indications of tubercular deposition. So she positively refused internal medication of any kind, except as a relief to her bowels. I simply gave her a discutent embrocation for the tumors, and the following mixture for her bowels:

℞ Ext. sennæ, f ℥ iij.

Tinct. jalapæ,

Tinct. gentian comp., aa ℥ iss. M.

A tablespoonful to be taken every night, and repeated in the morning, if not sufficient to produce a movement; and the dose to be gradually lessened as the bowels become regulated. Directions as to diet and hygienic measures generally were also laid down.

Mrs. K—— had been in Deavenworth one year, at the end of which time no one would have recognized her as the same woman. She had grown strong and robust, and the unfavorable symptoms had entirely disappeared. She again became pregnant. When about two months advanced, a fire occurred which consumed her dwelling, and for a period of one hour she was engaged with others in removing her household goods to a place of safety, and sat for the most of the night in the damp air. Notwithstanding our apprehensions, she recovered from the shock and its subsequent exposure and exhaustion, with no symptom of abortion.

About two months after this, while riding out with her husband after night, the buggy was overturned by a stump in the road, the occupants thrown out, and Mrs. K—— sustained a fracture of the humerus, and was obliged to walk in that condition a half mile to the nearest house. She recovered from this, much to her own surprise as well as ours, without bad symptoms, and in due time gave birth to a healthy boy.

Several other cases less marked could be cited, did space permit. I am aware that it may be urged that women in the last stages of phthisis, and other exhausting diseases, pertinaciously complete the term of pregnancy in opposition to many adverse circumstances, but I regard all such cases as being purely exceptional in their character. Probably no less prolific in producing a diseased ovum than the scrofulous and syphilitic taints, is that state of general cachexia and impoverished blood, produced by the excessive mercurialization, so extensively resorted to for the cure of syphilis, and in the West in the treatment of malarial diseases. This state of things, however, under a more prudent and enlightened system of medication, is begining to be much more rare, and it is to be hoped that the time is not far distant when empyrics shall cease to truthfully stigmatize the profession with the charge of curing one disease by the substitution of another infinitely worse.

Gentlemen of the association, if the foregoing remarks shall induce you, in cases of repeated abortion, where all probable and possible causes shall have been removed, to inquire more closely into the constitutional and hereditary tendencies of your patient, and treat her aborting proclivities by an attention to these circumstances, my end in making them will have been attained.—*Lancet & Observer.*

